# Foot Orthosis and Sensorized House Slipper by 3D Printing

**DOI:** 10.3390/ma15124064

**Published:** 2022-06-08

**Authors:** Lorenzo Brognara, Massimiliano Fantini, Kavin Morellato, Gabriela Graziani, Nicola Baldini, Omar Cauli

**Affiliations:** 1 Department of Biomedical and Neuromotor Sciences (DIBINEM), Alma Mater Studiorum University of Bologna, 40123 Bologna, Italy; lorenzo.brognara2@unibo.it; 2 Romagna Tech s.c.p.a., 47121 Forli, Italy; msm.fantini@gmail.com; 3 Department of Industrial Engineering, Alma Mater Studiorum, University of Bologna, Via Terracini 28, 40131 Bologna, Italy; morellato.kavin@gmail.com; 4 Laboratory of Nanobiotechnology, IRCCS Istituto Ortopedico Rizzoli, Via di Barbiano 1/10, 40136 Bologna, Italy; gabriela.graziani@ior.it; 5 Biomedical Science and Technologies Lab, IRCCS Istituto Ortopedico Rizzoli, Via di Barbiano 1/10, 40136 Bologna, Italy; nicola.baldini@ior.it; 6 Nursing Department, University of Valencia, Avda Menendez Pelayo 19, 46010 Valencia, Spain; 7 Frailty Research Organizad Group, University of Valencia, Avda Menendez Pelayo 19, 46010 Valencia, Spain

**Keywords:** gait, foot care, foot orthosis, mechanical properties, polymers

## Abstract

Background: In clinical practice, specific customization is needed to address foot pathology, which must be disease and patient-specific. To date, the traditional methods for manufacturing custom functional Foot Orthoses (FO) are based on plaster casting and manual manufacturing, hence orthotic therapy depends entirely on the skills and expertise of individual practitioners. This makes the procedures difficult to standardize and replicate, as well as expensive, time-consuming and material-wasting, as well as difficult to standardize and replicate. 3D printing offers new perspectives in the development of patient-specific orthoses, as it permits addressing all the limitations of currently available technologies, but has been so far scarcely explored for the podiatric field, so many aspects remain unmet, especially for what regards customization, which requires the definition of a protocol that entails all stages from patient scanning to manufacturing. Methods: A feasibility study was carried out involving interdisciplinary cooperation between industrial engineers and podiatrists. To that end: (i) For patient-specific data acquisition, 3D scanning of the foot is compared to traditional casting. (ii) a modelling GD workflow is first created to design a process permitting easy creations of customized shapes, enabling the end user (the podiatrist) to interactively customize the orthoses. Then, (iii) a comparison is made between different printing materials, in order to reproduce the same mechanical behavior shown by standard orthoses. To do this, the mechanical properties of standard materials (Polycarbonate sheets), cut and hand-shaped, are compared with four groups of 3D printed samples: poly(ethylene glycol) (PETG), poly(acrylonitrile-butadiene.styrene) (ABS), polycarbonate (PC) and poly(lactic acid) (PLA) obtained by Fused Filament Fabrication (FFF). Results: Differences found between the foot plaster model obtained with the plaster slipper cast in a neutral position and the model of the real foot obtained with 3D scanning in the same position can be ascribed to the non-stationarity of the patient during the acquisition process, and were limited by a locking system with which no substantial differences in the almost entire sole of the foot scan were observed. Conclusions: Using the designed GD workflow, podiatrists with limited CAD skills can easily design and interactively customize foot orthoses to adapt them to the patients’ clinical needs. 3D printing enables the complex shape of the orthoses to be reproduced easily and quickly. Compared to Polycarbonate sheets (gold standard), all the printed materials were less deformable and reached lower yield stress for comparable deformation. No modifications in any of the materials as a result of printing process were observed.

## 1. Introduction

Recent technological development gives health-care professionals including podiatrists a number of advantages and solutions to automate the manufacture of conventional orthoses. FFF 3D printing for the manufacture of anatomical orthosis leads to new production opportunities, which would otherwise not be feasible using traditional methods: the ability of 3D printing to use a unique material to achieve different densities by designing multi-scale hierarchical structures in the same thickness provides further manufacturing detail. For this reason, 3D-printing of custom-made orthoses has been largely pursued, and has recently been proposed for application in the podiatric field [1]. However, to date, only a few reports are devoted to this field, so the available solutions lack customization for the clinical need. Customization of the orthoses is particularly important for diabetic patients, for whom it would be possible to print customized insoles, optimizing the plantar pressure distribution by applying functional gradient structural properties to the insole with a different regional gradient modulus [1,2,3].

A recent study involving runners and patients with symptomatic flatfoot reported no significant differences in biomechanical or comfort parameters between customized 3D printed insoles and traditional plaster-molded foot orthoses [4]. In addition, 3D printer insoles were more effective than prefabricated insoles [5]. Substantial progress has been made in terms of manufacturing speed, the range of materials that can be used, the final product quality and reduced equipment cost, as a result of technological development compared to subtractive manufacturing [6,7,8,9,10,11,12,13]. The objective of the present study was therefore to assess the feasibility of a user-friendly and cost-effective solution to produce customized functional foot orthoses and sensorized house slippers, combining the use of low-cost devices for 3D scanning and 3D printing with an automatic modelling approach [14,15,16,17,18,19,20,21]. This is a step forward in creating a systematic and cost-effective protocol that will permit their customization to any patient and clinical need and boost their efficacy.

A growing number of papers are also reporting on the use of 3D scanning technology [22,23,24,25,26,27,28,29,30,31,32,33]. The promising role of 3D scanning and 3D printing technologies has been reported in several areas, including the manufacture of customized prosthetics and orthotics by scanning body parts, without any physical contact with the patient or interference in the measurement [34,35,36,37]. Compared to available literature, here, we combine all stages of the orthoses design, from custom-made scanning to manufacturing of the orthosis.

In the design processes, tools and methods are quickly evolving from Computer Aided Design (CAD) into Generative Design (GD), enabling the user to obtain complex design tasks by a semi-automatic modelling process, and to customize the resulting models.

To that end:

- For patient-specific data acquisition, 3D scanning of the foot was compared to traditional casting (plaster slipper cast in neutral position).
- A Generative Design (GD) workflow was developed to enable podiatrists to easily design and interactively customize foot orthoses and a sensorized house slipper.
- The most 3D printing materials commonly used for manufacturing customized foot orthoses were compared.

## 2. Materials and Methods

While the potential value of 3D scanning to obtain 3D images and models of an individual body part are becoming increasingly evident, various critical aspects in the clinical field remain to be determined. A comparison between the neutral suspension casting technique and scanning procedure, examining the accuracy of the negative mold, is a crucial point for clinicians to understand the differences between handmade and innovative methods. The cost of these devices is another factor that must be factored into the decision to use this technology, and, as such, a low cost 3D scanner (Sense 3D scanner; 3D System; Rock Hill, SC, USA) was chosen. In 2011, Carrol et al. investigated the reliability and accuracy of plaster casting and non-weight bearing laser scanning, and their results suggest that digital scanning is a reliable technique able to reduce measurement variability compared to neutral suspension casting [38,39,40,41].

### 2.1. Foot Scanning

**Figure 1:**
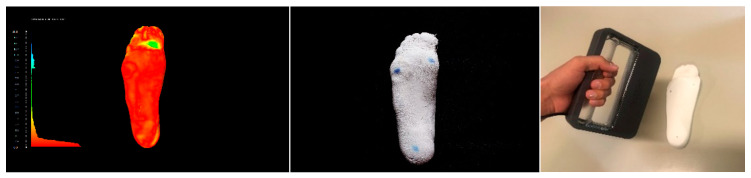
Comparison of ten plaster casts obtained after footprint recording with pinstripe and subtalar joint bandage in the neutral position (a neutral suspension cast).

An initial comparison was carried out by scanning ten plaster casts obtained after footprint recording with a pinstripe and subtalar joint bandage in a neutral position (gold standard). The ten plaster casts were uploaded into the open source software MeshLab (Visual Computing Lab–ISI—CNRresearch center; Pisa, Italy), version 1.3.3, and the Iterative Closest Point (ICP) algorithm was applied to automatically align the ten meshes (Figure 1). To better visualize error, the computed distance values were also visualized using a quality color filter. The casts have an error of under 0.74 mm (Table 1). The aim of this analysis was to compare the accuracy and reproducibility of the conventional approach [42].

**Figure 2:**
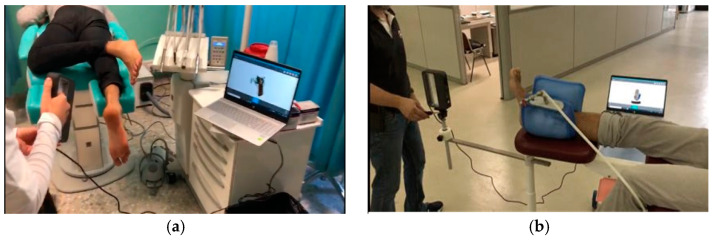
Scanning of the foot without any foot locking system and a handheld 3D scanner (**a**), and scanning of the foot with a foot locking system and a tripod for the 3D scanner (**b**).

The scanning of the plantar surface of the foot (direct approach) was carried out using a handheld 3D Sense scanner (Sense 3D scanner; 3D Systems; Rock Hill, SC, USA) without any foot locking system (Figure 2a), reporting an error of 1.16 mm (Table 2). A further comparison was therefore performed in order to validate a foot locking system. We used a vacuum silicon bag to set the foot in the corrected position (a neutral suspension scan) and a tripod for the 3D scanner to improve precision and reduce human error during the acquisition procedure (Figure 2b), and the error in this case was less than 0.84 mm (Table 3). The study results show that no substantial differences in the almost entire sole of the foot scan were observed compared with plaster casting, with the practitioner only using the locking system during the 3D scanning procedure.

### 2.2. GD Workflow for Orthosis Customization

**Figure 3:**
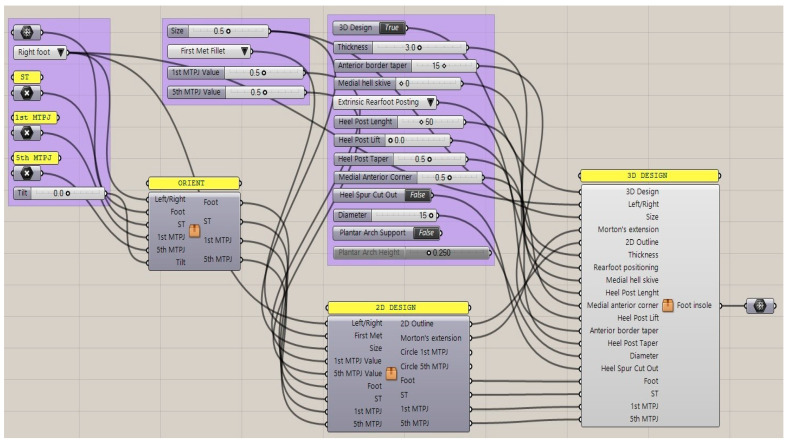
Generative design workflow with input data and sliders of the control panel for interactively modifying the foot orthosis and adding corrections (Grasshopper).

During the design process, we formalized a GD workflow that provided the specifications to generate a customized foot orthosis and sensorized house slipper that is specific to each patient’s anatomy according to their clinical needs. Moreover, this method allows for interactive modification of the geometrical features of the foot orthosis (shell thickness, heel size, etc.) by simply moving the sliders of the control panel and producing the watertight mesh ready for the next AM process in a semi-automatic approach. This approach enables the user to undertake complex design tasks in an automatic modelling process, and to customize the resulting geometrical models by interactively modifying certain parameters. In fact, there were insufficient CAD tools to take full advantage of AM in order to obtain the most common foot orthosis corrections in an intuitive way. The proposed workflow in Grasshopper is intuitive, and permits easy and interactive customization of the final foot orthosis (Figure 3). Moreover, this workflow could be modified and improved in order to semi-automatically design specific devices to meet patient demand and to design further developments in integration with electronic components for smart technology testing. The input data were the mesh of the scanned foot and three reference points, located in the first and the fifth metatarsophalangeal joints and the center of the calcaneus. The study investigated the feasibility of manufacturing intrinsic corrections, which are modifications to the positive mold and shape of the orthotics, using an automatic approach to enable health practitioners without enough CAD skills to easily design and use the anatomical modeling process.

**Figure 4:**
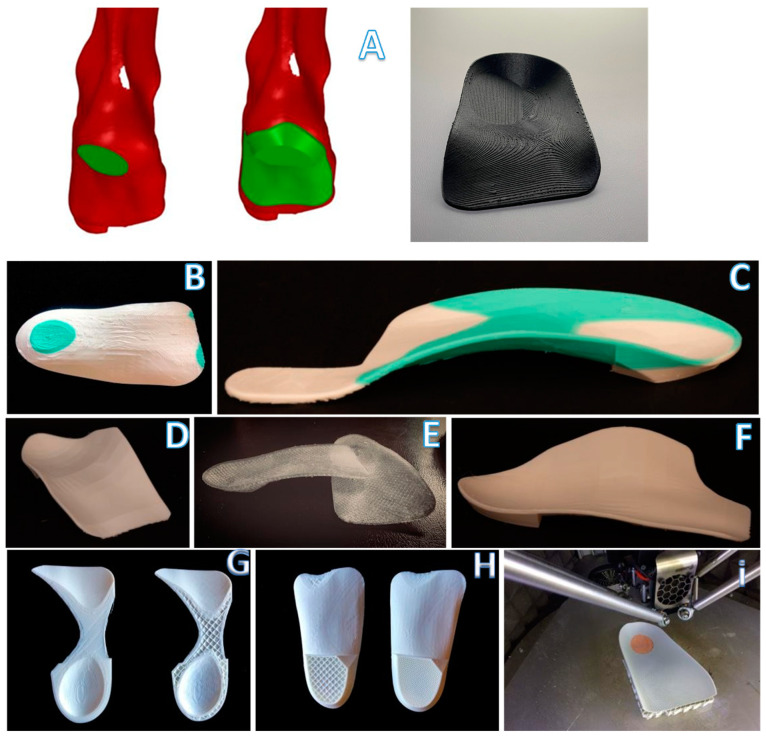
Key steps in the modelling process for the medial heel skive (**A**) and intrinsic corrections: intrinsic rearfoot post (**B**), Morton’s extension (**C**), first ray cut out (**D**), neutral orthosis without corrections (**E**), first met cut out (**F**) and 3D orthotics with different thicknesses and structure types (**G**–**I**).

The study selected some of the most common intrinsic corrections, such as Morton’s extension, first ray cut out, intrinsic rearfoot posting, first met cut out and medial heel skive (Figure 4A–F,). We also created a rearfoot post to stabilize the orthotic shell in a desired direction with the possibility of generating many different structures on the nanoscale level in order to have different densities (Figure 4G,H), thickness and materials, such as compressible filaflex, which will permit greater shock absorption (Figure 4I).

In contrast to the subtractive manufacturing process, rapid prototyping (RP) is a layer-by-layer additive manufacturing technology with similar accuracy and precision compared with the CNC and milling process [43,44].

The market currently offers many different machines based on this additive technique. FFF machines are split in two categories: Delta machines and Cartesian machines. This distinction is based on the need to move the extruder or the board to obtain route tracking layer by layer. For this application, we used a Delta Wasp FFF 3D printer operating as a delta robot. The FFF technique is based on the extrusion of heated feedstock plastic filaments through a nozzle tip. Different materials and nozzle sizes perform different deposition paths and model parameters. FFF is one of the most extensively used AM processes for fabricating orthoses inexpensively. The accuracy is about 0.05–0.30 mm, depending on the forms, geometry, orientation and printer [45]. The use of fine layers to build thickness and a large bead width increase both the surface quality and dimensional accuracy [46]. In terms of geometry and shapes, the output model obtained with this innovative technique demonstrates that this additive manufacturing technique has a comparable output to subtractive techniques.

Based on a layer-by-layer deposition, numerous limitations are present, such as imperfect matching between layers, incomplete filling and undefined mechanical torsional and tensile strength. This technique can be used for this specific insole application, where compressive loads are predominant.

The 3D model obtained by the GD process is then exported in an STL file to manufacture the customized foot orthosis [47,48,49,50]. In 2017, Miguel Davia-Aracil reviewed production cost overheads (such as machinery, materials, etc.) with an accurate feasibility and cost analysis and showed the advantages and disadvantages in cost terms of the two manufacturing methods used to produce foot orthoses. The author quantifies the production cost per pair of insoles obtained with the subtractive process as $31.92, and, for insoles obtained with an additive process for a production of 400 pairs per year, it is as $18.17.

### 2.3. Three-Point Bending Test

In terms of mechanical behavior, Polycarbonate specimens were used as the gold standard and its mechanical behavior is preferred for producing corrective insoles [51]. This led to the need to evaluate the mechanical properties related to common materials, obtained with different manufacturing techniques to better match the mechanical behavior of the gold standard material.

**Figure 5:**
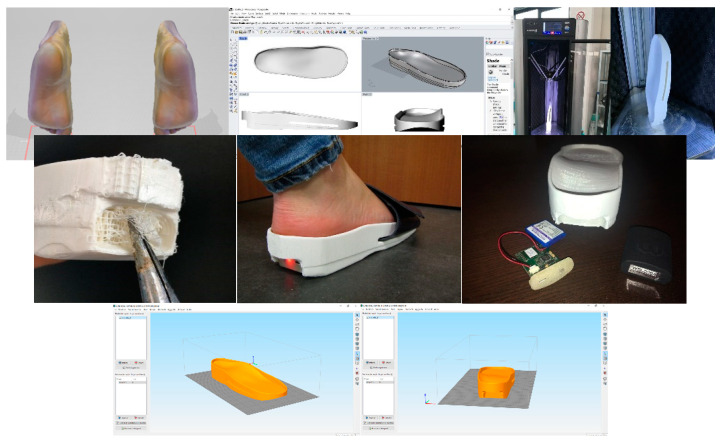
Key steps in the modelling process for the sensorized house slipper.

For the comparison of the basic orthosis material (Polycarbonate) used as “gold standard” with four groups of specimens based on FFF additive manufacturing techniques (Figure 5), each group was tested in a 3-point bending test. The four FFF specimen groups chosen in agreement with podiatrists are shown below:
*5 Polycarbonate Sheet*→(*control specimens*)*5 Polycarbonate Extruded*→(*printed specimens*)*5 ABS Extruded*→(*printed specimens*)*5 PETG Extruded*→(*printed specimens*)*5 PLA Extruded*→(*printed specimens*)

The first types of specimens were tested to assess the behavior of the control specimens, to compare the printed specimens and to understand the mechanical properties.

The test performed was a 3-point bending test from the control position. The actuator rate was set at 0.03 mm/s and the test continued until failure. The load cell used was an Instron 1 KN dynamic cell. The actual specimen dimensions were according to the ISO-178 test (the distance between the two supports is 64 mm and the specimen dimensions are: length 80 ± 2 mm; width 10.0 ± 0.2 mm; thickness 4.0 ± 0.2 mm). The force and displacement were recorded at 500 Hz throughout the test. The force–displacement plots were converted to engineering stress and engineering strain. When evaluating the mechanical properties of each specimen, we extracted the main parameters:

- The 1st Failure stress was defined as the first change of slope in stress/strain plot greater than 1% of the previous sample.
- The Max stress was defined as the maximum stress value measured throughout the test.
- The Young Modulus was computed with a linear regression of the stress–strain plot in the range between 20% and 80% of the 1st Failure stress.
- The 1st Failure Work was computed as the integral under the stress–strain curve until the 1st Failure stress occurs.
- The Total Work was computed as the integral under the stress–strain curve until the final failure.

### 2.4. FT-IR Test

The possible alterations caused by printing to the composition of the different materials were investigated by FT-IR ATR (PerkinElmer Spectrum 2, Waltham, MA, USA) acquisition parameters: resolution 4 cm^−1^, 16 scans, data interval 1 cm^−1^.

## 3. Results

### 3.1. Plot Overview

**Figure 6:**
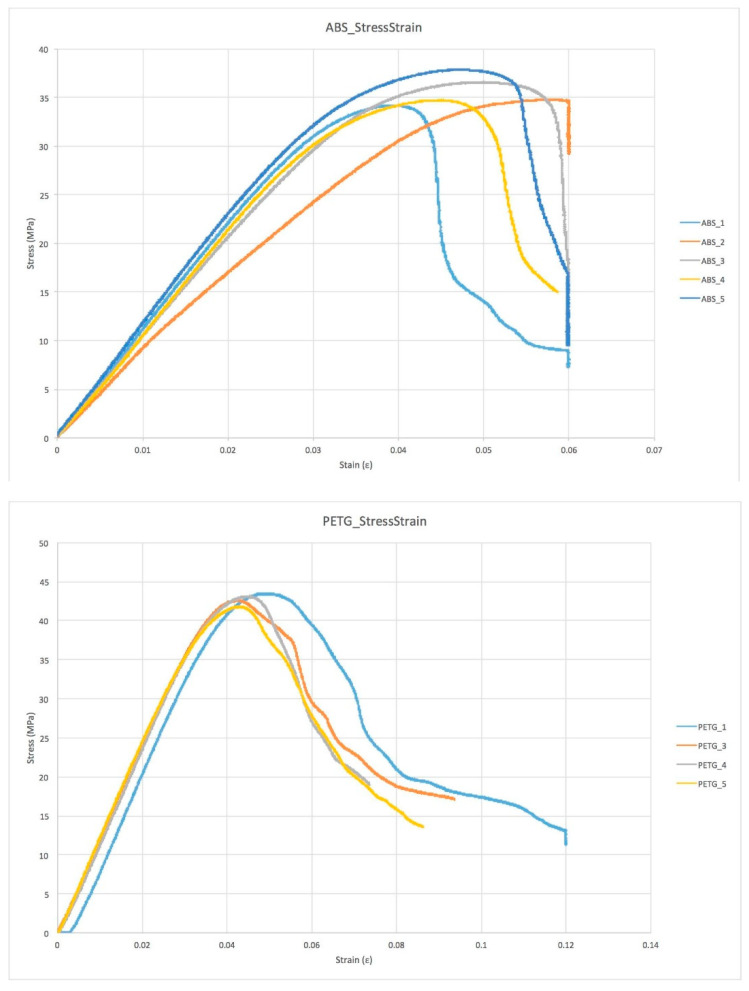

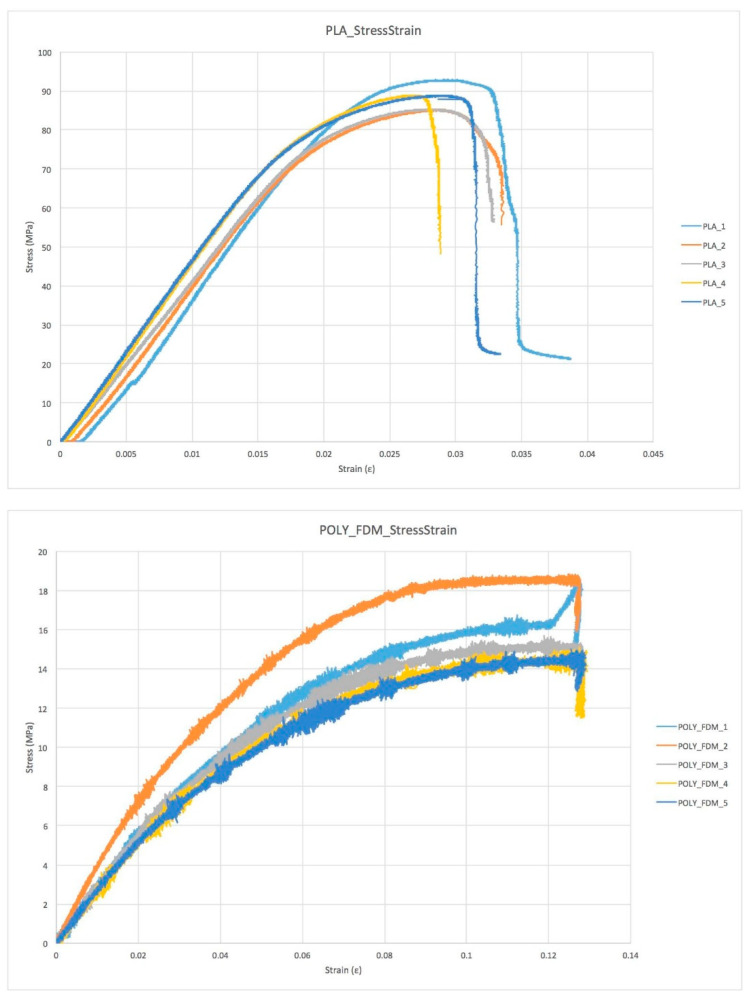

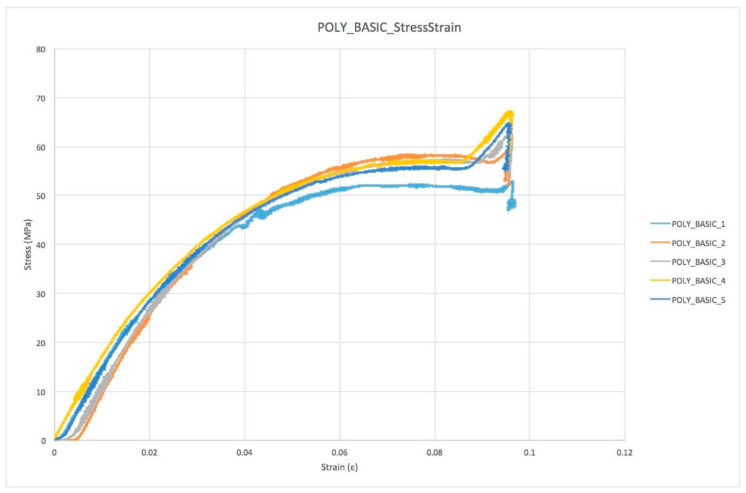
Stress/strain plots for the different sample groups.

The stress–strain behavior of the materials is highly homogeneous within each group, indicating a good reproducibility in the printing process, but it significantly varies between the different groups as a result of differences in the behavior of each material (Figure 6). During the mechanical test, the specimens were monitored visually to better understand the failure mode and the mechanical properties (Table 4).

### 3.2. Modes of Failure

**Figure 7:**
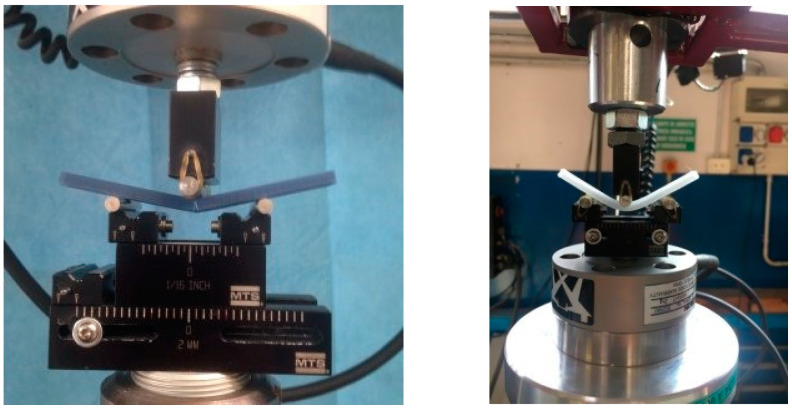
Set-up overview: (**right**) PC sample from sheet, (**left**) ABS sample, both from 3D printing filament material.

Different modes of failure occur during the mechanical test. As is evident (Figure 7), different materials present different modes of failure. ABS, PLA and PC (POLY_FFF) specimens obtained with additive manufacturing showed an extended plastic region compared to the PETG specimen. This was visible during the last second of the mechanical test. While the PETG specimen shows an instant break and crack propagation, ABS, PLA and POLY_FFF specimens show a slow crack propagation and breakages occur after a few seconds.

**Figure 8:**
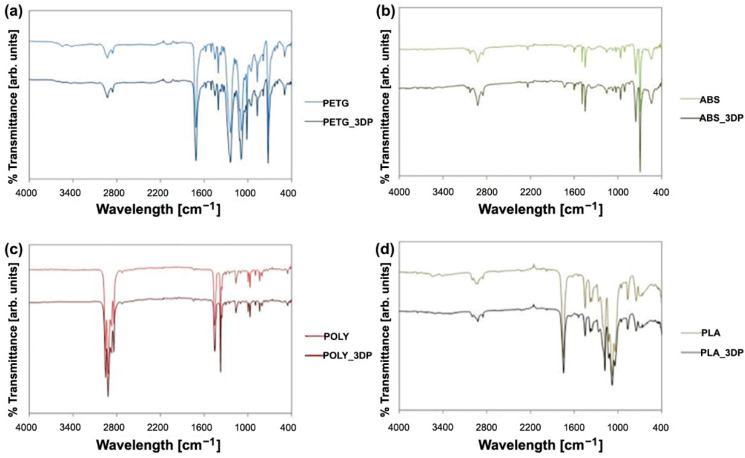
FT-IR bands of (**a**) PETG, (**b**) ABS, (**c**) PC, (**d**) PLA.

The results of FT-IR are shown in Figure 8. No modifications are assessed in any of the materials as a result of the printing process, as shown by the unmodified shape and position of all the FT-IR bands.

## 4. Discussion

The results of the present study show the effectiveness of a new approach for the production of orthoses based on a 3D scanning of the foot. We have demonstrated that the use of a low-cost 3D scanner with a locking foot system during the 3D scanning procedure is a feasible alternative to conventional procedures. Thanks to various automated software programs with dedicated protocol algorithms, we were able to identify the most effective process after visualizing errors and critically reviewing the production phases for the new additive manufacturing process, as well as conventional techniques through a self-objective analysis [52]. 3D scanning and related software enable the podiatrist to quantify the cast shape and orthotic treatment, thereby providing several potential improvements in foot orthotic manufacturing techniques.

In the digitizing process, the point cloud resulting from laser scanning in the low-cost system (Sense 3D scanner) seems to be reliable for the present practical purposes. However, the acquisition process has to be completed in a short time, and was more accurate since the patient has to be relaxed and their foot must be firmly fixed in the neutral position. This practice is less invasive and more comfortable for the patient, and is a cleaner process for the practitioner, while dramatically reducing material wastage. In the Generative Design process, the proposed workflow in Grasshopper is intuitive and enables the final foot orthosis to be easily and interactively customized. This workflow could also be modified and improved in order to semi-automatically design specific devices to meet the demands of patients with specific pathologies (i.e., hallux limitus, medial deviation of the subtalar joint axis).

An important topic for future research would be to investigate the different orientation of the 3D model in the printer volume, the different environments and the notch and bed temperature that influence the chemical bonds. This involves completely different behaviors under the same load condition.

Furthermore, thanks to new software updates, we were able to provide new information for a continuous improvement of the process. A Generative Design (GD) workflow was developed, and some feasibility tests involving Industrial Engineering and Podiatrists indicated that customized foot orthoses can be designed by a non-experienced practitioner in a very intuitive and interactive way. We believe an acceleration of the critical design review phase to be worthwhile as a key stage in the orthoses’ implementation process at which the design and development phase ends, and the manufacturing phase starts.

In terms of mechanical behavior, the use of a 3-point bending test was adopted, using the loading machine in displacement control in order to obtain a similar condition to human walking loads. Tests show different types of failure for different materials, a similar stress–strain plot trend for specimens of the same material and a large discrepancy from the gold standard material tested.

Both standard and printed polycarbonate specimens have similar strain values and types of failure. Printed specimens show lower levels of stress and work (due to an intrinsic specimen production process). The other materials are stiffer than the gold standard material and failure occurs quickly, and this can create a problem for the patient, in terms of both predicting failure and plantar injuries, and they are therefore clearly useless for podiatry purposes. In the FT-IR results, no modifications are assessed in any of the materials as a result of the printing process.

3D printed specimens are obviously structures with material gaps as a consequence of the additive technique used for the study. A different extruder path with higher spatial resolution would undoubtedly produce fewer gaps during filament deposition. On the other hand, the limitations remain strictly connected to the nozzle size and filament diameter. Other additive techniques best meet these two needs (Stereolithography SLA or Selective Laser Sintering SLS), but the material cost and safety management still remain a huge limitation for most podiatry centers.

An important topic for future research would be to investigate the different orientation of the 3D model in the printer volume, different environments and the nozzle and bed temperature that influence the chemical bonds. This involves completely different behaviors under the same load condition. Despite FFF technology being the most used for 3D printed orthoses and the most diffused technology available in small to medium-size clinics, in the future, a comparison between all 3D printing methods (SLA, Digital Light Processing DLP, Continuous Digital Light Processing CDLP, Multi Jet Fusion MJF, SLS etc) can be explored in terms of mechanical properties and printing limitations [53].

The proposed Generative Design workflow in Grasshopper allows the easy and interactive customization of final foot orthoses. Some feasibility tests involving medical staff showed that a customized foot orthosis can be designed by a non-experienced user in less than 5 min.

## 5. Conclusions

In the present paper, we reported a new protocol for customized foot orthoses manufacturing. The protocol is cost-effective and easy to employ for the clinicians.

As for the 3D scanning procedure, we have shown that the acquisition technique process was more accurate since the patient has to be relaxed and their foot must be firmly fixed in the neutral position compared to 3D scanning of the foot without any foot locking system and with a handheld 3D scanner.

As for printing, we have shown that, in terms of mechanical properties, the choice of the polymeric materials heavily affects the manufacturing process, so this aspect must be carefully evaluated by the clinician. At the same time, the different directions of loads in identical 3D printed models made in the same fashion in the building chamber of the 3D printer could react in different ways. Further tests need to be done in terms of both direction of printing and mechanical loading conditions in order to assess which materials best match daily life and medical needs. Since the market of 3D printing filaments is growing rapidly, further tests with different materials (both flexible and rigid) can therefore be performed to find the most effective.

The 3D digital approach opens up many new and exciting opportunities. However, a more detailed mechanical characterization is recommended, in order to assess the behavior of the orthoses after simulated ageing. In addition, future research in several areas (such as diabetic and rheumatic patients) must be performed in order for the technique to become clinically feasible. Each pathology affecting the foot requires different types of material (in terms of shore) based on the degree of disability and patient’s age. Clinical studies with large numbers of patients will indicate the best materials and techniques for each specific pathology and foot deformities.

## Figures and Tables

**Table 1:** Comparison of ten plaster casts obtained after footprint recording with pinstripe and subtalar joint bandage in the neutral position (gold standard).

Casts Obtained after Footprint Recording with Pinstripe and Subtalar Joint Bandage in the Neutral Position (Gold Standard)						
		Mesh-Mesh Alignment	Global Alignment	Hausdorff Distance (mm)		
Control Cast	Scanning of the Cast	Avg Err	Error Bound	Max	Mean	RMS
**1**	2	0.16	0.00	11.36	0.26	**0.69**
**1**	3	0.27	0.00	4.87	0.32	**0.48**
**1**	4	0.45	0.00	11.14	0.57	**0.86**
**1**	5	0.83	0.00	16.40	1.60	**2.49**
**1**	6	0.21	0.00	15.66	0.57	**1.35**
**1**	7	0.51	0.00	9.93	0.39	**0.95**
**1**	8	0.66	0.00	10.68	1.48	**2.18**
**1**	9	0.19	0.00	11.12	0.54	**1.27**
**1**	10	0.23	0.00	19.28	0.67	**1.92**
					**0.74**	

**Table 2:** Comparison of ten scans of the foot without any foot locking system and with a handheld 3D scanner.

Scanning of the Foot without Any Foot Locking System and with a Handheld 3D Scanner						
		Mesh-Mesh Alignment	Global Alignment	Hausdorff Distance (mm)		
Control Scan	Scanning of the Cast	Avg Err	Error Bound	Max	Mean	RMS
**1**	2	0.21	0.00	22.67	0.91	**1.29**
**1**	3	0.29	0.00	50.72	1.68	**4.64**
**1**	4	0.29	0.00	23.95	1.20	**3.06**
**1**	5	0.21	0.00	25.91	0.96	**1.17**
**1**	6	0.23	0.00	19.60	1.28	**1.23**
**1**	7	0.21	0.00	13.35	0.93	**1.51**
**1**	8	0.28	0.00	15.92	1.14	**1.75**
**1**	9	0.18	0.00	12.62	0.83	**1.04**
**1**	10	0.29	0.00	25.46	1.31	**3.46**
					**1.16**	

**Table 3:** Comparison of ten scans of the foot with a foot locking system and a tripod for the 3D scanner.

Scanning of the Foot with a Foot Locking System and with a Tripod for the 3D Scanner						
		Mesh-Mesh Alignment	Global Alignment	Hausdorff Distance (mm)		
Control Scan	Scanning of the Cast	Avg Err	Error Bound	Max	Mean	RMS
**1**	2	0.23	0.00	22.29	1.06	**3.21**
**1**	3	0.35	0.00	15.89	0.85	**2.83**
**1**	4	0.22	0.00	21.61	0.69	**2.05**
**1**	5	0.24	0.00	12.34	0.92	**2.12**
**1**	6	0.33	0.00	18.94	0.44	**3.25**
**1**	7	0.24	0.00	19.04	1.30	**2.66**
**1**	8	0.35	0.00	7.86	0.66	**1.12**
**1**	9	0.35	0.00	18.18	1.20	**3.22**
**1**	10	0.28	0.00	8.98	0.74	**1.13**
					**0.85**	

**Table 4:** Overview of all the relevant parameters obtained after the mechanical test and data processing from each sample group tested.

Specimen Group	Specimen n°	σ 1st Failure (MPa)	ε 1st Failure	Work 1st Failure (MPa)	σmax (GPa)	ε max	Work TOT (MPa)	Young's Modulus (MPa)
			
	**1**	32.1	0.032	0.562	34.3	0.060	0.948	1040.00
	**2**	32.0	0.031	0.412	34.9	0.060	0.456	795.59
**ABS**	**3**	32.1	0.034	0.575	36.6	0.060	1.044	977.04
	**4**	29.2	0.028	0.418	34.8	0.059	0.891	1050.90
	**5**	34.0	0.033	0.605	38.0	0.060	1.246	1062.10
	**Average**	31.9	0.032	0.515	35.7	0.060	0.917	985.13
	**Std Dev**	1.5	0.002	0.082	1.4	0.001	0.260	99.25
			
	**1**	88.0	0.023	1.019	92.9	0.039	1.693	1040.00
	**2**	78.2	0.028	0.847	85.2	0.033	0.943	795.59
**PLA**	**3**	80.0	0.021	0.917	85.4	0.033	0.941	977.04
	**4**	75.3	0.017	0.658	89.0	0.029	0.944	1050.90
	**5**	80.1	0.019	0.843	89.0	0.033	1.560	1062.10
	**Average**	80.3	0.022	0.857	88.3	0.033	1.216	985.13
	**Std Dev**	4.2	0.004	0.118	2.8	0.003	0.338	99.25
			
	**1**	42.1	0.042	0.900	43.6	0.120	2.248	1230.00
	**2**	42.0	0.043	1.072	42.7	0.094	1.619	1160.00
**PETG**	**3**	42.2	0.043	1.024	43.2	0.074	1.289	1198.00
	**4**	41.0	0.043	1.055	41.9	0.086	1.594	1139.00
	**Average**	41.8	0.043	1.013	42.9	0.094	1.688	1181.75
	**Std Dev**	0.5	0.000	0.068	0.6	0.017	0.349	34.97
			
	**1**	8.5	0.034	0.153	18.5	0.127	0.488	250.77
	**2**	10.7	0.034	0.206	18.8	0.127	0.719	300.02
**POLY_FFF**	**3**	8.5	0.036	0.172	15.7	0.127	0.496	238.88
	**4**	8.0	0.035	0.155	15.1	0.128	1.327	228.65
	**5**	8.2	0.037	0.167	15.0	0.128	0.448	218.65
	**Average**	8.8	0.035	0.171	16.6	0.127	0.696	247.39
	**Std Dev**	1.0	0.001	0.019	1.7	0.000	0.330	28.39
			
	**1**	50.4	0.027	0.513	53.0	0.097	1.584	1308.03
	**2**	55.0	0.024	0.331	59.6	0.096	1.540	1555.05
**POLY_BASIC**	**3**	55.2	0.027	0.435	63.0	0.096	1.444	1454.20
	**4**	55.1	0.027	0.547	67.3	0.097	1.468	1377.40
	**5**	55.0	0.026	0.483	64.9	0.096	4.027	1415.10
	**Average**	54.2	0.026	0.462	61.6	0.096	2.013	1421.96
	**Std Dev**	1.9	0.001	0.075	5.0	0.000	1.008	82.18

## Data Availability

The datasets generated for the present study are available from the corresponding author upon a reasonable request.
